# “I have surly passed a limit, it is simply too much”: women’s and men’s experiences of stress and wellbeing when living within a process of housework resignation

**DOI:** 10.1186/s12889-016-2920-5

**Published:** 2016-03-04

**Authors:** Lisa Harryson, Lena Aléx, Anne Hammarström

**Affiliations:** Department of Sociology, Umeå University, 901 87 Umeå, Sweden; Department of Nursing, Umeå University, Umeå, Sweden; Department of Public Health and Clinical Medicine, Epidemiology and Global Health, Umeå University, Umeå, Sweden

**Keywords:** Housework, Gender equality, Health, Wellbeing, Stress, Grounded theory, Masculinities, Femininities

## Abstract

**Background:**

Gender inequality within paid and unpaid work exposes women and men to different environments and responsibilities. These gender patterns shape living conditions for women and men, either negatively or positively, by affecting the prospect of good health. Most public health studies of gender and housework are quantitative, and knowledge about the relationship between housework experiences and health for women and men is limited. The aim of this study was to explore the housework experiences and practices of women and men and their experiences of stress and perceived wellbeing from a gender perspective.

**Methods:**

We conducted thematic interviews with four women and four men living in Sweden, and performed an analysis using the Grounded Theory method.

**Findings:**

We found that stereotypical gender practices in housework influenced experiences of stress and perceived wellbeing among women and men. Despite proposing gender equality in housework as a means of improving wellbeing, inequality was amplified by the way women and men handle the gendered division of housework. We call this recurring theme “The process of housework resignation”, which also constitute the core category in our analysis. “The process of housework resignation” was theorised from the categories “Gender practices in housework”, “Experiencing stress and wellbeing” and “Managing daily life”.

**Conclusions:**

Stereotypical gender practices in housework can increase experiences of stress among women and men. Challenging stereotypical masculinities can be a key for breaking the process of resignation in housework and for facilitating improved health among both women and men in heterosexual couple relationships within a Swedish context.

## Background

Women and men in Nordic countries are employed to almost the same extent, but work in various occupations and have different positions in the labour market [1]. Although Sweden is working towards a firm political goal of gender equality, it is well known that Swedish women perform most of the unpaid work in the home [2, 3]. Gender inequality within paid and unpaid work positions exposes women and men to different environments and responsibilities, and thus shapes their quality of life by affecting the prospect of good health either negatively or positively [4, 5]. Most public health studies on housework are also quantitative, and knowledge of the relationship between health and domestic work experiences is limited. For example, in a previous Swedish study we found that unequal division of housework was associated with psychological distress among both women and men, but the data did not allowed for exploring the social processes behind this association [6]. Furthermore, studies on this relationship among both women and men from a gender perspective are lacking. The aim of this study was to explore the housework experiences and practices among women and men and experiences of stress and perceived wellbeing from a gender perspective.

According to previous Nordic studies, combining work and family life can enrich one’s health, and the combined roles of employee, partner, and parent can protect against stress [7, 8]. However, combining work and family life can also contribute to conflicting demands between the two, which has proven to increase the risk of mental illness and physical symptoms [9, 10]. Additionally, a Swedish study has shown that it appears to be a critical point around which the workload resulting from combined waged work and housework could lead to either a decreased or increased risk of mental health problems [11].

Women who work fulltime and are primarily responsible for domestic work often experience stress and illness, and lack time to care for themselves [12]. In particular, mothers’ maintenance of their own health is inhibited due to numerous household and childcare responsibilities [13]. These results can be related to a previous Swedish study showing that women tend to experience higher degrees of housework-related stress than men, which is related to diminished feelings of wellbeing [8]. However, a heavy housework burden has also shown to correlate to mental illness among both women and men living in a Swedish context [6, 14].

Moreover, the perception of fair distribution of household responsibilities is important for the wellbeing and may contribute to a marital and family satisfaction meanwhile the opposite perception may cause conflict [14–16]. In a Swedish context, it has been shown that perceptions of fairness could potentially vary according to gender expectation. For example, if the social norm is that women perform the bulk of the domestic work, such a distribution of the work will more likely be perceived as fair [17]. Relationships that include perceptions of unfairness can produce risk of distress, whereas fair and supportive partners in intimate relationships can contribute to marital satisfaction and help to improve wellbeing and reduce distress [18, 19].

For this study, we viewed fairness in housework and marital satisfaction in heterosexual relationships as part of the construction of gender relations in everyday life [20–23]. An important theoretical concept from this perspective is the division of labour by gender, which describes the expectation that certain housework tasks are to be performed by women while others are to be performed by men [23, 24]. For example, unpaid work in the home is generally defined as women’s work regardless of men’s participation. Different notions of masculinities and femininities also exist within the social practices that produce and reproduce gender inequality in housework and health [23]. Previous studies has shown that a gendered division of housework have implications for the psychological well-being of both mothers and fathers of young children [6, 8]. There seems also to be a relational affect within heterosexual couples where men’s involvement in housework has the potential to decrease women’s risk of being distressed and unhappy [25].

Furthermore, housework is widely related to gender as a dominant principle of how society is organized and the gendered expectations we have of each other and ourselves. Thus the gender order, which describes larger societal gender patterns, is another important theoretical concept [23]. The gender order has been recognised as patriarchically divided, meaning that masculinity norms and practices are socially defined in contradistinction to femininities [26]. Since women to a greater extent are expected to work in the home and men in paid work, men have the advantage in terms of money, authority, respect, emotional support, control over one’s own life, and so forth [23]. These benefits can be important resources for maintaining good health, though there are differences and hierarchies among men [26, 27]. Overall, existing gender practices and expectations in everyday life can impact the conditions by which women and men may experience both stress and wellbeing [4, 23, 28, 29].

Our theoretical health perspective is based on the assumption that social circumstances and relationships with other persons substantially affect one’s health and wellbeing [18, 30]. We define health as individual’s physical, mental and emotional experiences [30]. From our view, health constitutes a dynamic and constant process that includes feelings of well-being, involvement, activeness, and reward in one’s everyday tasks. Thus we believe health and wellbeing is experienced rather than achieved [31]. Correspondingly, lack of wellbeing often depends on the individual’s subjective feelings about the world she (or he) creates for herself (or himself) and others, and can be seen as the self-perception of feeling ill [18, 31]. Stress, which may occur when demands from the environment exceed personal resources and thus endanger wellbeing, can contribute to illness. Both cognitive and emotional mechanisms can minimize lack of wellbeing resulting from stressful situations [32]. In situations when stress has occurred, resignation can be a way of ignore the stressor or being unable to change the situation despite how stressful it may be [33].

## Methods

### Setting

We conducted our study in a medium-sized town in northern Sweden with a gender-segregated labour market. Women occupy the majority of social services, education and care work positions, while men hold most of the jobs in construction, energy supply and manufacturing [34]. Two dominant discourses held in the Swedish society are that: firstly, the economic private sector has been de-politicized and there is a focus on providing free choices, and secondly, equality between women and men has already been achieved [35]. However, official statistics show another picture, highlighting that gender inequality is a structural rather than individual problem and that many areas of Swedish society still portray gender inequality. For example, although women are more highly educated and work to almost the same extent as men, they receive lower wages and perform the greater part of the unpaid domestic work [3]. The disparity between the discourses on already-achieved equality and actual inequality characterises the context of gender equality in Sweden today.

### Study design

We used a social constructivist Grounded Theory approach, which is a method of constant comparison particularly useful for analysing and illustrating social processes and complex factors that affect experiences of stress and wellbeing [36]. We used this method because it fits well with our gender theoretical framework in viewing gender as social constructed [22], and because it is useful for scrutinising the social processes involved in everyday life experiences [36]. Another reason for choosing this method is our intention to build a theoretical understanding emerging from the data.

### Participants

Four women and four men aged 47 years that are living in heterosexual couple relationships with children aged 6–18 years were interviewed. None of those who participated in the study was partner with another. The respondents were selected by previous participation in a health study called the Northern Swedish Cohort, a closed cohort of participants followed since 1981 [37]. Within this cohort, gender inequalities in housework has previously shown to be associated with psychological distress among both women and men, although deeper analyses of the social process that possibly could be explain some part of this association has not been analysed [6]. Based on the most recent survey of the Northern Swedish Cohort (conducted in 2008), we made a selection of participants who lived with a partner and children. Participants were also selected with the purpose to reach people in different situations of gender equality to increase opportunities for a rich empirical material. Therefore, selected participants to this study was those who had answered that they live in a gender equal relationship as well as participants who had answered that they did not live in an equal relationship. Selection also allowed for variation in gender and socioeconomic position. Among the respondents, the women worked with administration and in the health care sector while the men worked with administration and building construction. All men and two women worked full-time whereas two women worked part-time. Two women and one man were educated at the tertiary level, and the others at the secondary level.

### Data collection

Interviews were conducted in 2012. Initially, the principal investigator of the Northern Swedish Cohort (AH) contacted participants by telephone and provided them with information about the aim of the study and assurances of their voluntary participation, confidentiality, and privacy. Subsequently, all the interviews were performed by one of the authors (LH), contacted the participants by letter and telephone. The interviews lasted about 1 hour and were recorded and transcribed verbatim. The participants chose the interview location, and thematic interviews were conducted. The interview included questions about wellbeing and stress relative to: everyday life, housework, parenthood, leisure, and ideals of gender equality. The interviewer endeavoured to create an open atmosphere so participants could feel comfortable and free to talk about their experiences. Data collection ended when the gendered aspects of our theoretical analyses were considered saturated, which is described in more detail under the following paragraph about data analysis [36].

### Data analysis

As grounded theory is a method that enables simultaneous data collection and analysis, it was possible to analyse the social process connected to division of housework, wellbeing and stress meanwhile the data collection was ongoing, so-called abduction. The analysis was conducted according to Charmaz constructivist grounded theory approach [36]. The analytical process started with writing down *memos* including reflections, thoughts and ideas directly after each interview. Memos were also taken through each step of the analysis. The authors read the transcribed interviews and performed an *initial open coding,* meaning that lines and segments of data were given codes that were closely related to the data. The open coding was conducted both by using OpenCode 3.4, a free software program developed by Umeå University [38], and by manual coding. In this analytical step we compared our various coding, discussed and identified concepts and actions that described the informant’s experience and strived to grasp the participants’ perspectives, actions, and meanings [36]. In each line, sentence and paragraph we asked ourselves “What is this about? What social process is being referenced here?” After the open coding, we started the process of *selective coding,* which means that we were naming and labelling open codes that had something in common and was relevant to the aim in order to create comprehensive categories. This process representing a higher level of abstraction as we used our open codes to forming subcategories and categories. During this process, characterized by intensive discussions of how to interpret our results, we identified properties and dimensions of the core category and created a schema grounded in the data that represented how the categories was related to each other, an analytical step called *theoretical coding*. In this final part of the analysis, our intention was to grasp the main social process in housework which was built on the categories and related to stress and wellbeing. In this final analytical step, it was striking how similar these stories were in terms of gendered social process. Despite a selection of participants from a previous survey where they had reported gender equality in the couple relations as entirely equal or unequal, our analysis showed that the most common patterns was a gender traditional division of housework. Theoretically, this lead to a saturation in descriptions of gendered processes of housework related to stress and wellbeing. Examples of the analytical process are presented in Table 1 and the theoretical schema are presented in Table 2.

**Table 1:** Examples of meaning unit, codes, subcategory, category and core category

Meaning unit	Codes	Subcategory	Category	Core category
It is divided male-female, traditionally so to speak	Traditional division			
She cleans and washes mainly and I do the heavier stuff	Doing the heavy work			
I’m the one shovelling snow and painting and carrying	Doing the outdoor work	Living with stereotypical masculinities	Gender practices in housework	Living within a process of housework resignation
Sometimes I would rather not repair anything that breaks	Reluctant repair

**Table 2:** Main results of subcategories, categories and the core category

*Respondents gender*	Subcategories	Categories	Core category
*Men*	Living with stereotypical masculinities	Gender practices in housework	
*Women*	Living with the burden of domestic work		
*Men*	Feelings of stress from stereotypical masculinities	Experiencing health and illness	Living within a process of housework resignation
*Women*	Household obstacles to women’s health		
*Women, Men*	Escaping		
*Women*	Searching for strength	Managing daily life	
*Women, Men*	Challenging stereotypical masculinities		

### Ethics statement

The study was approved by the Regional Ethical Review Board in Umeå, Sweden. According to Swedish law, written consent is not required for this type of study [39, 40]. Participants gave their oral consent when they participated in the interviews.

## Results

The analysis resulted in the core category “Living within a process of housework resignation” which was built on the categories “Practising gender division in housework”, “Experiencing stress and wellbeing” and “Managing daily life”. The categories were built up by seven sub-categories (Table 2). The results are presented by category and thereafter, the core category is presented.

### Gender practices in housework

Both women and men practised a gendered division of housework, establishing a separation between women’s and men’s work at home. The described experiences of constantly being mainly responsible for repair and seasonal outdoor work as *living with stereotypical masculinities*. As men, they felt obligated to do certain tasks at home, some of which were related to their self-described role as the family’s “main breadwinner.” One man said, “I’m the one who shovels the snow, repairs things that are broken, paints the house when needed and carries heavy things. I often say that she [his partner] has me just for carrying, fixing and paying.” Among men there were explanations that the division of housework was based on expectations of their role as men, and was not a result of open negotiation.

Among women, a gendered division of domestic work caused feelings of *living with the burden of domestic work*. The women meant that they were mainly responsible for housework, and performed most of the everyday domestic tasks such as washing clothes and dishes, and cleaning the house. Their expressions included: “I have to wash all the time,” “others are just leaving things,” and “I do not want to come home from work and always clean up after others.” The division was not experienced as a result from an open negotiation but was based, instead, on practical reasons: that it just happened to be like this by chance, and that the tasks needed to be done regardless of who performed them. Financial dependence on the male partner further maintained the unequal division, as women who earned less money and/or worked part-time jobs felt obligated to perform most of the work at home.

### Experiencing stress and wellbeing

Both women and men described wellbeing as a subjective and emotional experience of feeling good, a feeling flowing in the background of their everyday life and the absence of perceiving illness and stress. Men expressed that when the family was all right, their own wellbeing was good. They also expressed that their wellbeing was good compared to men of similar age, or to their own health history. But when narrating about own daily life the men experienced *feelings of stress from stereotyped masculinities* due to expectations of the man to always solve problems and repair things. They described feeling drained, stressed, and inadequate because of too many things to do. One man said: “I have done this for so long now so I have surely passed a limit. It is simply too much.” “Passing the limit” included experiences of lacking energy and sleeping poorly because of work-home pressure. There was also descriptions of feelings of stress when the gender equality ideal was inconsistent with practices at home.

Women expressed that wellbeing was: feeling a “tingle in the heart,” mediating your knowledge and experiences with others, being active, and feeling useful. However, the gendered practices in housework contributed to *obstacles to women’s wellbeing.* For the women, constantly cleaning up after others was accompanied by constant feelings of stress and worries. They described insufficient sleep due to anger at having to clean and wash late in the evenings. One woman said: “I cannot sleep when I’m angry because of the domestic work.” There were also expressions of a need for perceiving wellbeing to manage the burden of domestic work. The women felt that they could not be exhausted when coming home from the job as there was a lot of domestic work that had to be done. One feature of experiences of stress was bad conscience, which the women experienced when the family wanted more attention than they could manage to give. Women also felt stressed when they were constantly disrupted by the family because it made it difficult to have some time of their own. They described a lack of dignity and self-esteem related to feelings of being forced to do the domestic work with no appreciation from the partner, which contributed to a lack of energy.

### Managing daily life

Women and men who practiced gendered housework managed their daily lives by *escaping* and engaging in other activities on their own. *Escaping* involved: physical activity, time with friends, creative activity, and development of new skills. They considered these activities relaxing and energizing. As another means of escape, both women and men also avoided discussions about the housework which they meant often led to quarrels, irritations, stress, and no change in practice. Women mostly preferred to do all the housework themselves rather than to start a discussion. One woman stated: “It is more like you feel that you’re getting nowhere. It [the discussions] is just irritating and does not lead anywhere. … It is not the chores themselves, because they need to be done anyway. It is rather that others are just leaving tasks undone, and then I have to take care of it myself.”

Among men, one way of escaping was by denying the presence of gender inequality in order to live an easy and privileged life at home without any housework. When the pressure from work and home was, nevertheless, was too high, men used alcohol to cope with the feelings of stress. One man said: “You drink more alcohol when you are stressed … it is a way to relax, I suppose.” Setting housework limits for themselves also helped to reduce stress among men. However, men believed that they should handle stress by themselves and without the help of others.

Another means by which women managed daily life was by *searching for strength* within themselves and believing that the situation would be solved in the future. Women also found strength from friends who made them feel important and took them seriously. One of the women said: “She [the friend] saved my life. I have told her that as well … she has a lot of empathy and she can talk so one really can see things from a different perspective. She was a life-saver, truly amazing.”

The category *challenging stereotypical masculinities* represented one way to break up gendered practices in housework among women and men. The participants noted that positive approaches by men to learning traditionally feminine housework practices could minimise quarrels at home. Challenging stereotypical masculinities was also a way for men to handle the fear of being stuck in a fixed masculine roles with limited opportunities to live the life they wanted. Modifying the division and sharing tasks equally between partners was also considered key for decreasing stress and balancing the workload throughout the year. One man explained: “The ideal would be that we shared everything equally. I think I would benefit from that in some sense; it would be rather a relief, actually. An equal share— that is my medicine”.

Women also wished for a more equal share of the everyday work, and for more of a contribution to the work from their male partners. However, women also experienced resistance from their partners when they suggested modifying the division. For example, one the women with university education said “I have tried to do lists, that we [she and her husband] could tick off so we can see who is the one doing the most. But it doesn’t work; it has not been well-received [by the husband]”.

### Living with the process of housework resignation

Despite proposing gender equality in housework as a means of improving wellbeing, both women and men continued their daily lives with unequal division of domestic tasks. For example, escaping the situation or searching for strength did not change the housework distribution but enabled the gender inequality to continue. Participants frequently described feelings of weariness and disappointment because of an apparent impossibility to achieve any change in the division of housework. We call this recurring core category living with the *process of housework resignation*. Feelings of ambivalence among women, who considered their domestic responsibilities both self-evident and unfair, were also associated with this theme. Given previous unsuccessful attempts to adjust the unequal division, participating women were uncertain whether their partner would ever contribute more to the housework. Thus, they felt they had to accept an unequal share. Men, on the other hand, blamed their female partner for unwillingness to perform some of the male-associated tasks and emphasized this point as the main obstacle to change. We understood that women had more limited ability overall to change the situation because of asymmetrical power relations and economic dependence on their male partner, while men were better able to choose between change and resignation. Concomitantly, men admitted that they sometimes had the privilege of periods with less housework while women felt constantly responsible for cleaning up after others, causing them to feel exhausted.

## Discussion

We identified a process of housework resignation in which stereotypical gender practices in housework were related to experiences of stress and perceived wellbeing among women and men. Despite proposing gender equality in housework as a means of improving well-being, inequality was amplified by the way women and men handled the gendered division of housework.

### Resignation, stress and wellbeing

Resignation refers to a cognitive escaping process and/or a passive attempt to ignore or avoid stressors [33]. Therefore, resignation could be a means of avoiding stress while hoping that time will take care of the situation, and/or passively accepting a situation despite how stressful it may be. In some situations where the source of stress is experienced as impossible to change, resignation can be a way of managing experiences of stress in the short-term [33]. From our theoretical health perspective, the process of resignation can be interpreted as a method of coping with unequal housework that causes feelings of stress and deteriorated well-being. In our study, resignation may have been depicted by women and men avoiding discussions about housework and engaging in activities on their own outside the family. Our findings about men’s denial of the importance of gender equality and use of alcohol as means of escape, and women’s feelings of ambivalence between self-blame and unfairness may also represent resignation in housework [33].

In our study, living with the process of housework resignation was the core category and resignation was one way used by women and men for managing daily life and handling experiences of stress. A previous study suggests three additional concepts for eliminating distress caused by conflicts at home: direct action, help-seeking, and positive thinking [41]. However, our analysis also showed that the women and men tried to tackle the housework by setting limits and making lists, respectively. Their attempts exemplified individual actions aimed at eliminating the stressor (*direct action*). Expressions of an appreciation about the idea of men learning feminine tasks were connected to willingness of trying to make such changes along with their partners (*help-seeking).* Direct action and help-seeking are behaviour-focused attempts to use control to solve the problem at hand. In addition to these strategies, there were experiences among women in our study to manage the high burden of housework in their daily lives by searching for strength from friends and believing in themselves. Their approaches demonstrate increased control of daily life, and cognitive and emotional responses that were optimistic (*positive thinking*).

Nonetheless, resignation was the most common way of handling the unequal division of housework among the participants in our study. This might result from ineffective attempts at direct actions, or an enduring resistance to implementation of the changes, like the men of our study assuming feminine tasks, because of strong gender norms. Although resigning can reduce distress, the strategies of direct action, help-seeking, positive thinking could probably have a more positive effect on wellbeing [33]. One reason for this could be that resignation often represents the abdication of control, which can make individuals perceive greater conflicts. Perhaps this perception arises because there is no change in behaviour, and therefore the issue continues to worsen silently.

Our results also indicate that failure to manage the gendered division of housework may also cause feelings of being “out of control,” which in turn may further exacerbate the experience of inequality. Research on stress management generally highlights the importance of psychological control and self-efficacy for effectively handling stress and illness [33]. In our study, women were more limited overall in their ability to implement change in the home, compared to men. Furthermore, applications of resignation and avoidance as means of stress management correlate to more perceived conflicts between work and family life [41]. Resignation as a way to deal with inequality in housework may contribute to a constant, never-ending conflict, potentially leading to many more problems for long term health.

### Women, femininity and stress

There were expressions of stress among women characterized by feelings of anger, exhaustion, ambivalence, bad conscience, distress, sleeping problems, and lack of energy. We found that the strong norms of women’s family responsibilities as wives and mothers contributed to a central part of constructing femininity, perpetuating a dependency of their male partners and increasing marital dissatisfaction. This corroborates previous findings that women are often entangled in a compliant private life and feel the normative pressure to primarily live up to be good mothers and wives [42, 43]. Our findings suggest that stress in women can be related to high amounts of work, unfair division of housework, and limited ability to change the inequality. Resignation adds to the experience of stress and can contribute to poorer psychological health [44]. In accordance with previous research, our results suggests that women who have lost the power to control their lives also suffer from a damaged self-esteem [43]. We found that gender structures in housework appears to negatively impact the lives of women by limiting their autonomy and causing experiences of stress and feelings of not being well. The women in our study also expressed an ambivalent in their satisfaction with the division of housework, and often felt they did more than what they considered a fair share, causing their wellbeing and marital satisfaction to deteriorate. This might be connected to previous studies showing that when one’s ideas of fairness in the organization of housework deviate from the actual experience, there is a risk of feeling deprived [45]. We conclude that for the participants in our study, the gendered divisions of housework entails that the supportive and health-protective function of the couple relationship is compromised for women, and that gender power asymmetries create obstacles to these women’s wellbeing.

### Men, masculinity and stress

None of the men in our study were primarily responsible for everyday housework. Nevertheless, the gendered division of housework was related to experiences of stress in men who were responsible for the burdensome, stressful, male-associated tasks. There were descriptions of stereotypical ideals and expectations of male-associated housework were unfavourable; we designated this experience “living through stereotypical masculinities.” It was emphasized that experiences of constantly fixing and repairing for others passed the limit for what was deemed healthy. However, since configurations and norms about masculinities are established through social practices within cultural, contextual and historical settings, they are subject to change [26]. The maintaining of a stereotypical masculinity should be contrasted with the ideals of “the new man” who renegotiates and engages in everyday domestic responsibilities [46]. Men in our study who challenged stereotypical masculinities were capable of empathising with their partner’s position, visualising the power inequality in the couple relationship and experiencing marital satisfaction. Previous Swedish studies has shown that such an approach could remove the frequent justification of unequal division of housework with excuses such as personal preference, financial responsibility and special circumstances [47, 48]. In the long run, this approach could be one way of increasing men’s opportunities to change their involvement in the home and thus possibly improve their wellbeing. Such a reasoning is in line with a cross-national study showing that the more involved men are in childcare and housework the higher they report family satisfaction [16].

### Gender and health on large scale

To understand the psychological, emotional and physical consequences of inequality in housework, it is important to recognise that the practice of gender relations within families and amongst couples is related to large-scale societal patterns [5]. Sweden is a country steadily working towards gender equality and have strong dual-earner ideals [2]. In such a context, we found that women in a position of being economically dependent on men contributed to their primarily responsible for everyday housework, while men felt a pressure to carry out stereotypically masculine tasks. It is possible that these gendered power asymmetries in the domestic sphere interrelate with gender inequalities on the labour market and hinder women and men from achieving abundant health.

#### Reflections on methodology

Study participants with various experiences and perspectives were selected from a longitudinal cohort study. Despite being selected as having varied views on gender equality within their couple relationships in the questionnaires, the participants in the interviews mainly described experiences of practicing gender inequality in household responsibilities. Among both women and men, there was an open criticism of own gender inequality in the couple relationship and a conscious effort to strive for gender equality in housework as a way of reducing stress and high demands, something that is not commonly found in previous research. A critical aspect of this results among men might be a social desirability towards the interviewer in combination with a societal pressure of a dominating discourse in the Swedish society to be positive to gender equality. On the other hand, it reflect a previous patterns found in a Swedish study where men express their support to gender equality in theory, but are not willing to change their behaviour in practice, which enables a proceeding of gender inequality in the domestic sphere [48]. Our interpretation is that the participants very openly shared their experiences during the interviews, answered our questions with great engagement and often brought up different aspects of gender equality and health themselves, which contributed to extensive and detailed information about their everyday lives. One reason for the openness among our participants might be related to the fact that the interviews did not only concern housework and gender equality but also health experiences. Also, the participants were interviewed individually, which may have made it easier for them to express critical aspects of the couple relationship than if their partner was present. It is also possible that the openness is a results of an interview situation characterised by the participants’ feeling comfortable about reflecting and sharing their experiences of gender inequality. This might be related to previous participation in the Northern Swedish Cohort that can have contributed to a trusting belief in the importance of their participation in research and the importance of freely sharing their experiences. Furthermore, in accordance with Charmaz [36], we view the interview as a mutual construction depending on the relation between the interviewer and the participant. Thus, the participants’ perceptions and experiences were constructed in relation to the interviewer and represent a particular point of view under certain circumstances. Variation in status and power between the interviewer and the participants, such as professional status, gender, age and ideology, can have an important influence on what can be said and how things can be communicated [49]. In this situation, it might be an advantage that the interviewer was a 30 year old Swedish women without own children that sincerely and curiously asked about how they viewed their life and asked them about their life experiences.

Although there were relatively few participants in our study, the selection process from a longitudinal cohort study and the rich content of the interviews contribute to the credibility of the study. Our use of Grounded Theory, which enables simultaneous data collection and analysis, further ensured study credibility. Moreover, the authors discussed the selective and theoretical coding in depth to achieve a high level of consistency. The data was systematically checked and the analysis and interpretation were constantly monitored and confirmed [50]. The analysis has also been presented at a Swedish national conference for work and family. However, critical aspects of our study was that it only included four women and four men and that the participants represented a relatively homogeneous group as they were 47 years old native born Swedish living in heterosexual couple relationships together with children in Sweden, a country that officially supports gender equality. This context must be considered for eventual transferability. Although our study contributes with describing an important gendered process of everyday life and wellbeing, future research need to include additional dimensions including ethnicity, age and/or sexuality in order to increase the knowledge of the gendered process of the complex relations between housework, stress and wellbeing.

## Conclusions

Although methodological limitations temper the transferability of the conclusions, the results from this study suggests that a process of stereotypical gender practices in housework can lead to experiences of stress among both women and men. In a Swedish context, resignation resulting from the gender unequal division of housework can be fuelled by maintained stereotypical ideals about masculinities and women’s limited possibility to implement change. It is possible that challenging stereotypical masculinities could be a key for breaking a gendered process of resignation in housework and for facilitating improved wellbeing among both women and men in Swedish heterosexual couple relationships.
